# The African Swine Fever Virus Transcriptome

**DOI:** 10.1128/JVI.00119-20

**Published:** 2020-04-16

**Authors:** Gwenny Cackett, Dorota Matelska, Michal Sýkora, Raquel Portugal, Michal Malecki, Jürg Bähler, Linda Dixon, Finn Werner

**Affiliations:** a Institute for Structural and Molecular Biology, University College London, London, United Kingdom; b Pirbright Institute, Pirbright, Surrey, United Kingdom; c Institute of Molecular Genetics, Czech Academy of Sciences, Prague, Czechia; d Institute of Healthy Ageing, Department of Genetics, Evolution and Environment, University College London, London, United Kingdom; University of Illinois at Urbana Champaign

**Keywords:** African swine fever virus, NCLDV, RNA polymerases, RNA-seq, gene expression, promoters, transcription, transcription start site, virology, zoonotic infections

## Abstract

African swine fever virus (ASFV) causes hemorrhagic fever in domestic pigs, presenting the biggest global threat to animal farming in recorded history. Despite the importance of ASFV, little is known about the mechanisms and regulation of ASFV transcription. Using RNA sequencing methods, we have determined total RNA abundance, transcription start sites, and transcription termination sites at single-nucleotide resolution. This allowed us to characterize DNA consensus motifs of early and late ASFV core promoters, as well as a polythymidylate sequence determinant for transcription termination. Our results demonstrate that ASFV utilizes alternative transcription start sites between early and late stages of infection and that ASFV RNA polymerase (RNAP) undergoes promoter-proximal transcript slippage at 5′ ends of transcription units, adding quasitemplated AU- and AUAU-5′ extensions to mRNAs. Here, we present the first much-needed genome-wide transcriptome study that provides unique insight into ASFV transcription and serves as a resource to aid future functional analyses of ASFV genes which are essential to combat this devastating disease.

**IMPORTANCE** African swine fever virus (ASFV) causes incurable and often lethal hemorrhagic fever in domestic pigs. In 2020, ASF presents an acute and global animal health emergency that has the potential to devastate entire national economies as effective vaccines or antiviral drugs are not currently available (according to the Food and Agriculture Organization of the United Nations). With major outbreaks ongoing in Eastern Europe and Asia, urgent action is needed to advance our knowledge about the fundamental biology of ASFV, including the mechanisms and temporal control of gene expression. A thorough understanding of RNAP and transcription factor function, and of the sequence context of their promoter motifs, as well as accurate knowledge of which genes are expressed when and the amino acid sequence of the encoded proteins, is direly needed for the development of antiviral drugs and vaccines.

## Abstract (precis)

African swine fever virus (ASFV) causes incurable and often lethal hemorrhagic fever in domestic pigs. In 2020, ASF presents an acute and global animal health emergency that has the potential to devastate entire national economies as effective vaccines or antiviral drugs are not currently available (according to the Food and Agriculture Organization of the United Nations). With major outbreaks ongoing in Eastern Europe and Asia, urgent action is needed to advance our knowledge about the fundamental biology of ASFV, including the mechanisms and temporal control of gene expression. A thorough understanding of RNAP and transcription factor function, and of the sequence context of their promoter motifs, as well as accurate knowledge of which genes are expressed when and the amino acid sequence of the encoded proteins, is direly needed for the development of antiviral drugs and vaccines.

## INTRODUCTION

African swine fever virus (ASFV) is the sole characterized member of *Asfarviridae* (1), a family resembling others in the group of nucleocytoplasmic large DNA viruses (NCLDV) and *Megavirales* order (2, 3). *Asfarviridae* also include the uncharacterized Abalone asfarvirus (NCBI taxonomy ID 2654827), while the faustoviruses show similarity to ASFV but have larger genomes and infect amoeba (Vermamoeba vermiformis) (4). ASFV originated in east sub-Saharan Africa where it remains endemic; it crossed continents to Georgia in 2007 (5), and its subsequent spread in Europe and to Asia in 2018 (6) has resulted in the current emergency situation. ASFV has a linear double-stranded DNA (dsDNA) genome of ∼170 to 194 kbp encoding ∼150 to 170 open reading frames (ORFs). Genomic variation between strains predominantly originates from loss or gain of genes at the genome termini among members of multigene families (MGFs) (7). Despite the global economic importance of ASFV, little is known about ASFV transcription, but it is believed to be related to the vaccinia virus (VACV) system (8–10), a distantly related NCLDV and *Poxviridae* family member (11).

We have focused our analysis on the BA71V strain (170,101-bp genome, with 153 annotated ORFs) (12, 13) because this is the most well-studied ASFV strain regarding viral molecular biology, including gene expression and mRNA modification (10, 14). Based on a paradigm of the vaccinia virus, several stages of ASFV gene expression have been hypothesized in the literature, including immediate early, early, intermediate, and late genes (10, 15–17). However, the experimental evidence for four discrete gene expression stages in ASFV leaves room for improvement though the presence of two alternative subsets of transcription initiation factors strongly supports the notion of at least two discrete stages, early and late, likely at pre- and postreplicative stages of the virus life cycle. Previous individual gene expression studies have made use of chemical inhibitors to inhibit replication or protein synthesis (10, 15, 16). While these are valid tools when used with care (18), the application of these chemicals is not unproblematic due to the possibility of indirect pleiotropic effects. For example, the nucleotide analogue cytosine arabinoside (AraC) can be incorporated into DNA, and while at low concentrations it mostly inhibits replication, it can interfere with the action of many DNA-binding enzymes, including RNA polymerases (RNAPs) and transcription factors as well as topoisomerase (19). In light of this, in this study we chose to characterize transcription unadulterated by chemical inhibitors.

ASFV inhabits the eukaryotic cytoplasm and appears to be self-sufficient in terms of transcription and modification of viral mRNA. It encodes an RNAP, a poly(A) polymerase, and an mRNA capping enzyme; importantly, extracts obtained from mature virus particles are fully transcription competent (10, 20, 21). The basal ASFV transcription machinery resembles the eukaryotic RNAPII system encompassing an (8-subunit) ASFV-RNAP and distant relatives of the TATA-binding protein (TBP), the transcription initiation factor II B (TFIIB), and the elongation factor TFIIS (8, 9, 13). ASFV also encodes a histone-like DNA binding protein, pA104R, and ASFV topoisomerase II (pP1192R), which collaborate to generate DNA-binding and supercoiling activity (22). Of particular interest is the possibility that the ASFV-RNAP gains promoter specificity in terms of temporal (early or late) gene expression, dependent on the association with either TBP/TFIIB-like or virus-specific factors including those encoded by ASFV BA71V genes D1133L and G1340L, which are homologous to the D6 and A7 (respectively) early transcription factor (ETF) heterodimer (23, 24) from VACV. Promoter consensus motifs for early and late ASFV genes have not been characterized on a genome-wide scale or in great detail, with the exception of an AT-rich sequence motif upstream of the p72 gene transcription start site (TSS) and some other late genes, as well as a consistently AT-rich region overlapping the TSS (25). Importantly, information about the temporal ASFV gene expression, the TSS, and the transcription termination site (TTS) is not available (10, 11).

**FIG 1:**
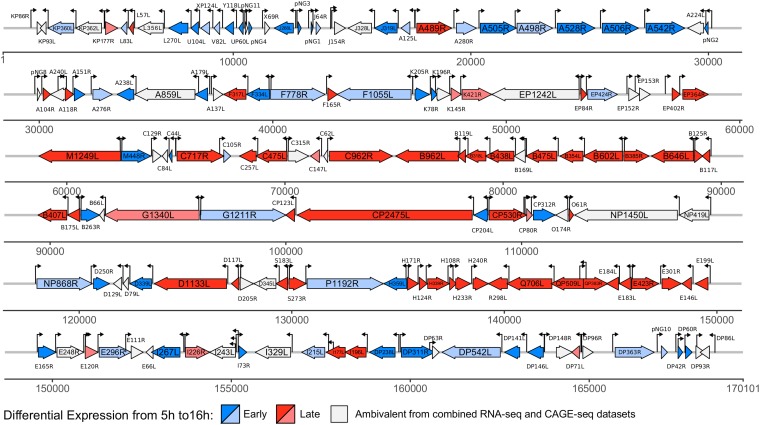
Annotated genome of ASFV-BA71V indicating transcription start sites (TSS) and early and late genes. The map includes 153 previously annotated genes as well as novel genes identified in this study and their differential expression patterns from early to late infection from DESeq2 (80) analysis. Early genes (upregulated, highlighted in dark blue) and late genes (upregulated, dark red) were differentially expressed according to both RNA-seq and CAGE-seq approaches. The pale blue and pale red markings indicate negative (early, downregulated) and positive (late, upregulated) log_2_ fold changes, respectively, in expression levels according to both CAGE-seq and RNA-seq data, but the change is statistically significant (adjusted *P* value < 0.05) only for data from CAGE-seq due to its higher sequencing depth; unlike RNA-seq, CAGE-seq is not affected by transcription readthrough. Ambivalence of early and late expression patterns (i.e., not statistically significant according to either of the methods or only according to RNA-seq) is also indicated. This group also includes 10 genes with reversed differential expression between CAGE-seq and RNA-seq results. The map was visualized with the R package gggenes.

We have applied a combination of next-generation sequencing (NGS) techniques including transcriptome sequencing (RNA-seq), RNA 5′-end cap analysis gene expression sequencing (CAGE-seq), and RNA 3′-end sequencing (3′ RNA-seq). We report (i) the ASFV transcriptome map showing differences in gene expression levels between early and late infection, (ii) a genome-wide TSS map that has allowed us to define early and late ASFV promoter consensus motifs as well as 5′ mRNA leaders, and (iii) a genome-wide TTS map that provides novel insights into the mechanism of transcription termination in ASFV. Figure 1 is a genome-wide map visualizing our results from TSS mapping and differential gene expression in ASFV.

## RESULTS

### Overview of the ASFV transcriptome.

A transcriptome is defined by the overall expression levels of transcripts and their 5′ and 3′ termini. We carried out RNA-seq, CAGE-seq, and 3′ RNA-seq in order to characterize these parameters during early and late ASFV infection; when the methods are combined, they provide information about the ASFV transcriptome and DNA sequence signatures associated with transcription initiation and termination. The processed data are compiled in an assembly hub and can be publicly accessed in the UCSC Genome Browser (available at https://bit.ly/2TazQxK).

**FIG 2:**
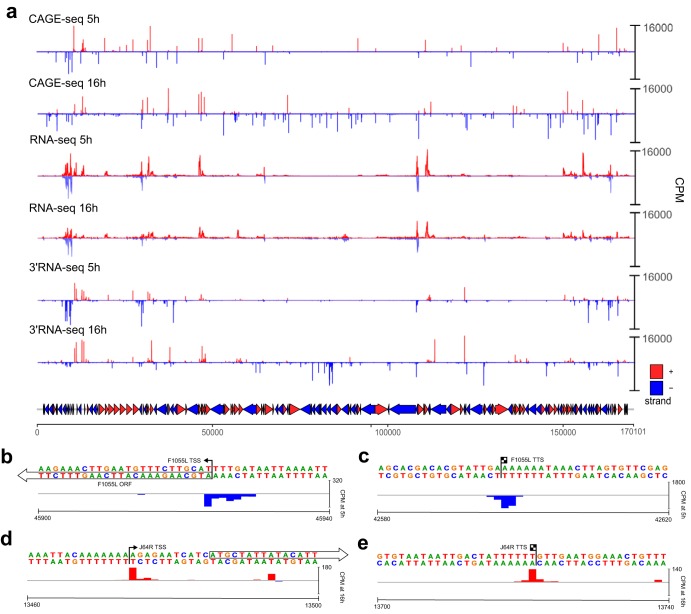
The ASFV transcriptome including transcription start sites and termination sites. (a) Whole-genome view of normalized coverage counts per million (CPM) of RNA-seq, 5′ CAGE-seq, and 3′ RNA-seq reads. The coverage was capped at 16,000 counts per million. A total of 153 BA71V annotated ORFs are represented as arrows and colored according to strand. Peak cluster shape examples are from F1055L 5′ CAGE-seq ends (b) and 3′ RNA-seq ends (c), showing a wide multipeaked distribution, and from J64R 5′ CAGE-seq (d) and 3′ RNA-seq (e), showing a narrow peak distribution.

Vero cells were infected with BA71V, and viral RNA was extracted at 5 h and 16 h postinfection (p.i.). These time points were chosen based on a previous report of a small subset of genes that were experimentally characterized using nuclease S1 mapping and primer extension analysis (10, 26). Bowtie 2 (27) mapping of the RNA-seq, CAGE-seq, and 3′ RNA-seq reads (summarized in Table S1 in the supplemental material) showed a strong correlation between replicates (Pearson correlation coefficient, *r *≥ 0.9), with the exception of RNA-seq data from 16 h (*r* of 0.74 and 0.84 for two strands [data not shown]). Figure 2a provides a whole-genome view of mapped reads from all three next-generation sequencing (NGS) approaches, while a selection of individual examples of TSSs and TTSs at single-nucleotide resolution is shown in Fig. 2b to e. The sequencing depth of the RNA-seq approach was more than sufficient to determine significant changes in ASFV transcription (i.e., reads) at early and late infection due the small genome size (170 kb). The majority of CAGE-seq reads (i.e., TSSs) were located upstream and proximal to ORF start codons. A subset of late-infection TSSs mapped to more distant locations between ORFs or within ORFs; these are caused by pervasive transcription, mRNA decapping and degradation followed by recapping, or BA71V genome misannotations (28–31). The increased background of TSSs was more noticeable during late infection (Fig. 2a, CAGE-seq 16 h) and was likely due to pervasive transcription, a phenomenon that has been observed in humans (32) and in VACV (28). The cause of this low-level and genome-spanning transcription is unclear but has been attributed to an open chromatin structure in cellular organisms (33). In viral genomes, it may reflect differences between nascent, newly replicated genomic DNA during late infection and genomic DNA still associated with histone-like proteins (such as A104R) just released from the virus particle during early infection.

### Mapping of ASFV primary transcription start sites.

**FIG 3:**
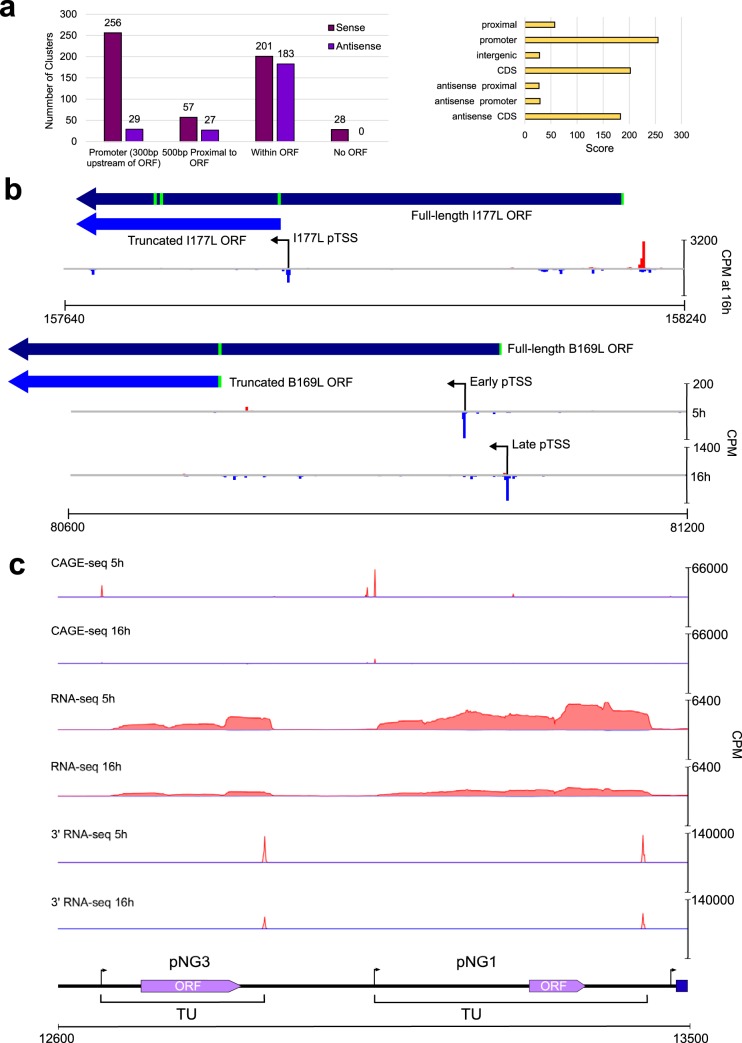
Transcriptome mapping aids the reannotation of the ASFV BA71V genome. (a) A summary bar graph (left) shows CAGEfightR TSS clusters and their locations relative to the 153 annotated BA71V ORFs. Types of CAGEfightR clusters detected and the distribution of their respective CAGEfightR scores are shown on the right. (b) Two examples of ORFs requiring reannotation following pTSS identification downstream of annotated start codon, encoding shorter ORFs from the pTSS (I177L, above) or during one expression stage (B169L, below). (c) Examples of two putative novel genes (pNG3 and pNG1) annotated with the normalized RNA-seq and CAGE-seq read coverage (counts per million [CPM]) and their genome neighborhood.

Following mapping of CAGE-seq reads to the ASFV BA71V genome, we located regions with an enrichment of reads corresponding to the 5′ ends of transcripts and thereby the TSS. We detected 779 clusters of CAGE-seq signals, and CAGE-seq clusters upstream of annotated ORFs were manually investigated to confirm that they represent primary TSSs (pTSSs) based on peak height, proximity to the ORF initiation codon, and coverage from our complementing RNA-seq data. We identified pTSSs fulfilling these criteria upstream of 151 BA71V ORFs; thus, only two genes, E66L and C62L, were not found associated with a pTSS. Overall, our data showed good agreement with previously individually mapped TSSs of 44 ORFs (Table S2). Not all of the ∼780 clusters were located within 500 bp upstream of ASFV ORFs but were within, or in the antisense orientation relative to, ORF coding sequences (Fig. 3a). We reannotated 11 ORFs based on gene-internal TSS and RNA-seq reads (Table 1; Fig. 3b, I177L). We provide a novel gene feature file (GFF) based on our revised annotations (see the supplemental material).

**TABLE 1:** Summary of ASFV genes for which pTSS locations guided the reannotation of ORFs

ORF	Strand^*a*^	pTSS coordinate (nt)^*b*^	Corrected start (nt)^*b*^	ORF length (aa)	Comment
K93L	−	2131	2122	83	Alternative ATG codon 30 nt downstream; another strong TSS was detected at nt 2037, whose transcripts would encode a 36-aa protein
F165R	+	42354	42359	136	Alternative ATG codon 63 nt downstream
C84L	−	64618	64492	38	ORF in frame with original C84L start codon
			64616	76	ORF encoded from first ATG after the pTSS
G1211R	+	96370	96377	1207	Alternative ATG codon 12 nt downstream
CP204L	−	108573	108567	196	Alternative ATG codon 24 nt downstream
CP312R	+	110491	110501	307	Alternative ATG codon 15 nt downstream
I177L	−	L: 157857	157849	66	Strongest pTSS detected only in a late time point
DP93R	+	167971	167980	83	Alternative ATG codon 30 nt downstream
EP402R	+	56862	57104	115	Encodes 115-aa in frame with original EP402R start codon
			56991	148	Alternative ORF encoded from first ATG after pTSS
B169L	−	80983 (E)	81018	169	
		81025 (L)	80745	78	Late pTSS can produce full-length B169L and early pTSS
I243L	−	155122 (E)	155119 (E/I)	243	
		155124 (I)			
		155115 (L)	154969 (L)	191	Late pTSS produces shorter transcript with closest downstream ATG encoding a shorter protein

a Plus (+) and minus (−) strands are indicated.

b For B169L and I243L, the letters E, I, and L refer to alternative pTSSs from early, intermediate, and late infection, respectively; for I243L, they are as reported by Rodríguez et al. (26).

**FIG 4:**
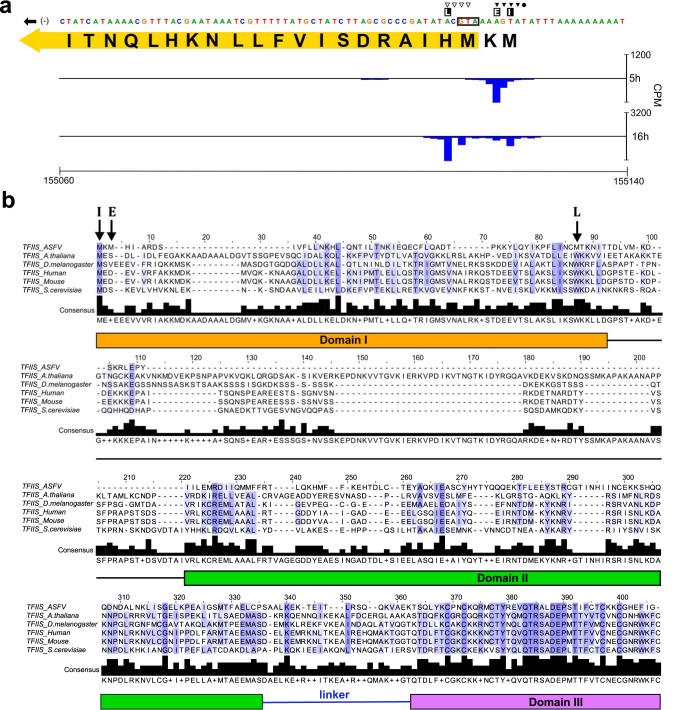
Analysis of alternative pTSS usage in I243L. (a) Close-up of TSSs (CAGE-seq alignments) on the minus strand at the start of the I243L ORF. Symbols indicate the TSS sites for early (▼), intermediate (•), and late (▽) gene expression according to Rodríguez et al. (26), while E, I, and L indicate, respectively, early, intermediate, and late gene pTSS positions concluded from our data. The first 21 aa residues of the annotated I243L ORF are shown; in yellow is the reannotated ORF which could be encoded in transcripts initiating from both of our annotated early pTSSs. (b) ClustalW multiple-sequence alignment colored by percentage identity between sequences at the same position from white (0%) to blue (100%), according to their agreement with the consensus sequence found below the alignment ('+' indicates positions where more than one residue is found in the modal consensus), illustrated with Jalview (84), of TFIIS homologues from ASFV (I243L; NCBI accession no. P27948), Arabidopsis thaliana (Q9ZVH8), Drosophila melanogaster (P20232), human (P23193), mouse (P10711), and Saccharomyces cerevisiae (P07273). S. cerevisiae TFIIS domain locations according to Kettenberger et al. (85) are shown below the alignment, and acidic (DE) catalytic residues are in domain III. ASFV-TFIIS start codons encoded from alternative transcription start sites are labeled as in panel a.

Several genes have a bona fide pTSS upstream of the annotated start codon and an alternative TSS residing within the included J64R (Fig. 2d) and B169L (Fig. 3b). The alternative downstream TSS of J64R is weaker than the upstream pTSS and specific to 16 h p.i. Our genome-wide CAGE results are confirmed by previous analysis of individual genes such as I243L (26), which was shown to have distinct TSSs for different stages of infection (Fig. 4a). I243L encodes a homologue of the polymerase II (Pol II) transcript cleavage factor TFIIS that is highly conserved between archaea and eukaryotes and among NCLDV members, albeit with limited domain conservation (34). TFIIS has dual functions: it stabilizes transcription initiation complexes and reactivates stalled elongation complexes by transcript cleavage (35, 36). The late TSS is located downstream of the I243L start codon, and the utilization of the next methionine codon would result in a TFIIS variant lacking 52 N-terminal amino acid residues (Fig. 4b). While the early and long transcripts encode the fully functional three-domain TFIIS factor, the late and short transcripts encode a truncation variant lacking the N-terminal domain that is responsible for initiation functions of TFIIS. In essence, the TFIIS variants expressed during early and late infection would have a different functionality. We identified seven further genes with alternative pTSSs during early and late infection (Table 2). In most cases, the reannotated (single pTSS downstream of start codon) or alternative pTSSs (multiple pTSSs, some downstream of the start codon) did not substantially alter the ORF protein products, except for reannotated I177L and alternative pTSSs of B169L, two putative transmembrane proteins (Fig. 3b) (13, 20).

**TABLE 2:** Alternative pTSS usage during early and late ASFV infection

Gene	Early pTSS position (nt)	Late pTSS position (nt)	Function (reference)
X69R	11315	11280	Uncharacterized
J154R	14174	14150	MGF 300-2R
EP1242L	53125	53135	ASFV-RPB2
C315R	70137	70131	ASFV-TFIIB
CP80R	110208	110191	ASFV-RPB10
D345L	129357	129257	Lambda-like exonuclease (7)
E120R	150949	150911	Structural protein (88)

### Novel genes supported by sequencing data.

Twenty-eight TSSs in our CAGE-seq data set were not associated with annotated ORFs (Table S3), and seven of these pTSSs were associated with transcripts that encode short ORFs, which we call putative novel genes (pNGs). These encode polypeptides 25 to 56 amino acids (aa) long that were missed in the initial BA71V ORF prediction as only ORFs of ≥60 aa were annotated (13). Five pNG ORFs showed limited similarity to short ORF-encoding genes from other ASFV strains, while pNG5 showed no clear similarity (Table 3). Interestingly, pNG6 was homologous to KP93L, which is already encoded by BA71V but barely expressed according to our data. In contrast, pNG6 was highly expressed at 5 h (Table S4). Figure 3c illustrates the features of pNG1 and pNG3, with distinct TSSs and TTSs and robust RNA-seq read coverage across the entirety of both genes. All pNGs had the same orientation as neighboring downstream genes (Fig. 1), and five of the seven pNG transcripts terminated promptly, i.e., were associated with a drop of reads following a 5- to 8-nucleotide (nt) thymidylate sequence (Fig. 3c) (10, 16). All of these observations support the notion that these transcription units (TUs) are new bona fide genes.

**TABLE 3:** Details of seven novel ASFV candidate genes^*a*^

Putative gene	Strand^*b*^	Transcription start site (nt)^*c*^	Transcription end site (nt)^*d*^	Putative protein length (aa)	Similarity according to NCBI BLAST		Gene-end no. of Ts
					Homologous sequence (accession no.)	E value	
pNG1	+	13053	13435	25	13 residues had 92% identity to ASFV-G-ACD-00350 (AZP54308.1)	0.11	6
pNG2	−	30091	29827	50	50 residues had 100% identity with ASFV26544 00600 (AKM05534.1)		8
pNG3	+	12664	12896	44	38 residues had 59% identity to ASFV-G-ACD-00290 (AZP54130.1)	0.13	6
pNG4	+	10583	10835	44	42 residues had 65% identity with ASFV-G-ACD-00290 (AZP54130.1)	1e−09	6
pNG5	+	29817	30080	31	No significant similarity		None
pNG6	+	167005	167336	56	56 residues aligned with 40% identity to pKP93L (AIY22188.1)	6e−07	5
pNG7	+	10484	10616	32	32 residues aligned with a 31-aa hypothetical protein with from ASFV Belgium 2018/1 with 87% identity (VFV47940.1)^*e*^	8e−10	3

a NCBI ORFfinder and BLAST were used to predict the putative encoded ORFs which were subsequently analyzed for putative homologous sequences (88, 89).

b Plus (+) and minus (−) strands are indicated.

c Defined as a pTSS from CAGE-seq analysis.

d Defined from 3′ RNA-seq analysis. Underlined transcription ends were defined from only RNA-seq. pNG5 is in the antisense orientation relative to pNG2, and the RNA-3′ end of pNG6 is dispersed according to RNA-seq and may overlap DP42R. pNG7 overlaps pNG4 on the same strand.

e From BioProject PRJEB31287.

### Highly expressed ASFV genes during early and late infection.

**FIG 5:**
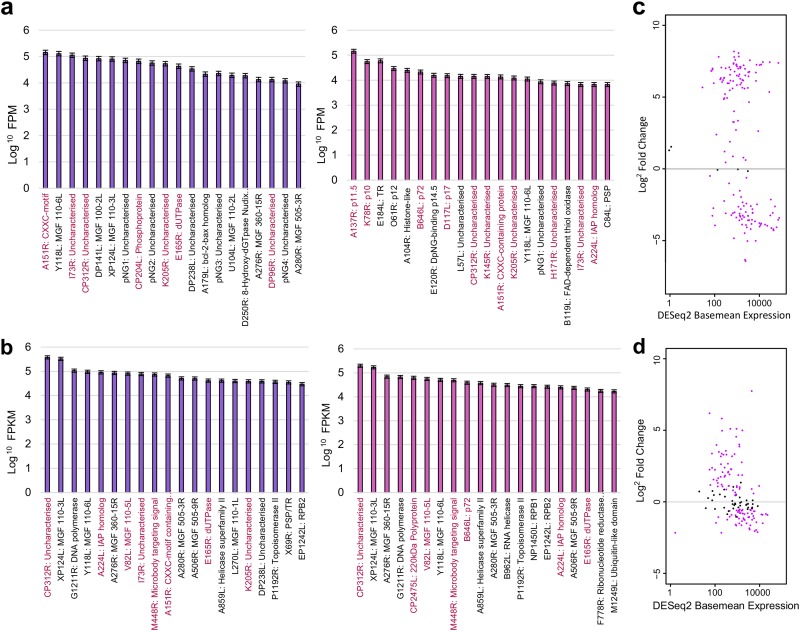
Gene expression of ASFV genes during early and late infection. (a) Fragments per million (FPM) values for 20 most highly expressed ASFV TUs according to CAGE-seq at 5 h (left) and 16 h (right) postinfection. Genes highlighted in dark pink indicate those encoding proteins which were also found in the 20 most abundantly expressed ASFV proteins during infection of either WSL-HP, HEK293, or Vero cells according to proteome analysis done by Keßler et al. (37). Gene functions are shown after the gene name with TR and PSP referring to predicted transmembrane region and putative signal peptide, respectively. (b) The 20 most expressed genes during early (green) and late (blue) infection according to RNA-seq data over gene TU, defined from TSS to ORF stop codon. (c) MAplot from DESeq2 analysis of CAGE-seq representing the DESeq2 baseMean counts of transcript levels versus their log_2_ fold change, with significantly differentially expressed genes in pink (adjusted *P* value of <0.05). (d) MAplot representing expression of ASFV TUs including pNGs from DESeq2 analysis of RNA-seq data.

In order to gain insights into expression of individual genes, we quantified mRNA levels obtained by CAGE-seq and compared the most abundant mRNAs at early and late time points (Fig. 5a). Table S4 summarizes expression of all detected ASFV BA71V genes, including the newly annotated pNGs. For this purpose, we temporarily redefined ASFV gene transcription units (TUs) as regions spanning from the pTSS to the stop codon (as a proxy for TTS; see below) and quantified TU expression based on RNA-seq data (Fig. 5b and Table S5), which closely reflected the CAGE-seq analysis. The highly expressed genes matched those identified in the viral proteome of infected tissue cultures determined by mass spectrometry (highlighted in Fig. 5a and b) (37). Six genes in the top 20 highly expressed genes were common during early and late infection (CP312R, A151R, K205R, Y118L, pNG1, and I73R). While their expression decreased from early to late infection (see below), these genes were clearly expressed throughout, suggestive of a multistage expression pattern. Considering their high levels of expression, they are likely important throughout infection, which makes them interesting candidates as potential drug or vaccine targets. However, four (out of six) have an unknown function (Fig. 5a) and await functional investigation.

### Differential expression of early and late ASFV genes.

**FIG 6:**
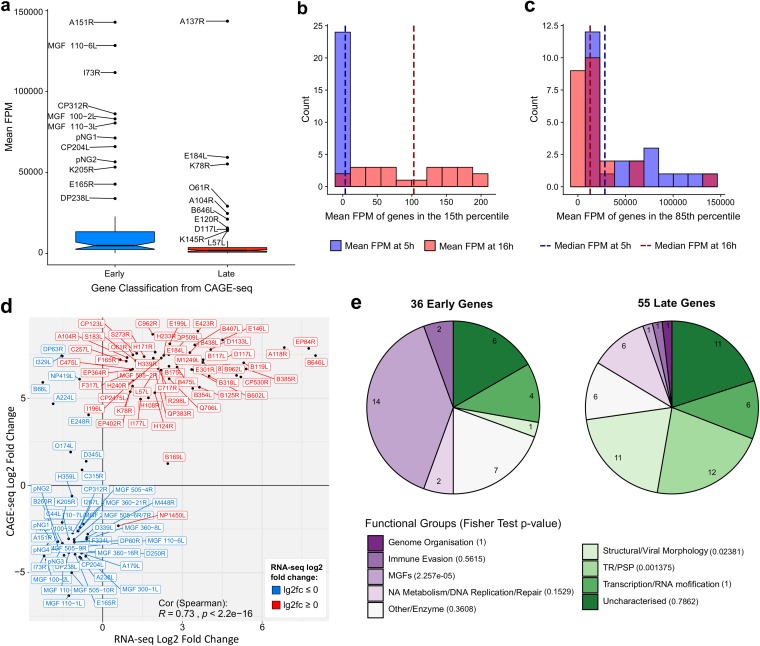
Relative expression during infection stages and defining early and late genes. (a) Box plot mean FPM values for the early and late genes at early and late infection, respectively. Outliers are labeled with their gene names. Wilcoxon rank sum tests showed that the mean FPM values of early genes during early infection was significantly greater than that of late genes during late infection (*P* value of 1.865e−06). (b and c) Distribution of the least and most expressed genes during early and late infection. Genes in the 15th percentile for their mean FPM values from each time point represent those below an early FPM threshold of 7.56 (blue) and late FPM of 199.64 (red). Genes in the 85th percentile for their mean FPM values from each time point represent those above an early FPM threshold of 8148.91 (blue) and late FPM of 4706.27 (red). In dark blue and dark red are medians for the plotted expression values for early and late infection, respectively. (d) Scatter plot comparing log_2_ fold changes of the 101 significantly differentially expressed genes in common between RNA-seq and CAGE-seq data. Labels were colored according to their significant upregulation or downregulation from RNA-seq data. (e) Pie charts of gene functional categories downregulated from 5 h to 16 h (36 early genes) and upregulated from 5 h to 16 h (55 late genes). Fisher’s test was carried out on gene counts for functional groups between early and late infection; for this all MGF members were pooled into the MGFs functional group.

We characterized differential expression of ASFV genes between early and late infection by comparing separate DESeq2 analyses of CAGE-seq and RNA-seq data sets (Fig. 5c and d, respectively). Based on RNA-seq data, 103 ASFV TUs showed significant differential expression (adjusted *P* value of <0.05), with 47 genes downregulated and 56 genes upregulated during the progression from early to late infection. Henceforth, we focused on the CAGE-seq data set because the reads are associated with the nascent transcription start sites and thus cannot arise from transcription readthrough from upstream genes (unlike mRNA quantification using RNA-seq), which would complicate the analyses. RNA-seq also has the disadvantage of a lower sequencing depth and thus lower apparent sensitivity than that of CAGE-seq. Indeed, CAGE-seq identified 149 genes as significantly differentially expressed, with 65 downregulated genes and 84 upregulated genes (Fig. 5c). Naturally, this is not a binary classification; i.e., genes that are upregulated during late infection do not have zero reads during early infection and vice versa. Interestingly, the relative expression levels of early genes at 5 h p.i. appeared significantly higher than those of late genes at 16 h p.i. (Fig. 6a). This is due to normalization of the reads and the increase of steady-state levels of all transcripts during late infection, which can be seen from the sequence alignment rates (Table S1). While the number of reads mapping to early genes during early infection is lower than that of reads mapping to late genes during late infection, the total number of reads mapping to all ASFV genes is higher during late infection. The per-gene fragments per million (FPM) values and differential expression analyses are normalized for ASFV-mapped sequencing depth, which therefore reduces this background and emphasizes highly expressed genes during early infection. Overall, we did observe a greater and cleaner contrast in expression of the genes during early infection than during late infection. The expression levels of the least expressed genes at 5 h p.i. were more consistent and closer to zero than those at 16 h p.i. (Fig. 6b). The most highly expressed genes at both time points were more similar, though relative expression of the most expressed genes at 5 h p.i. was higher than that at 16 h p.i. (Fig. 6c). In summary, it appears that ASFV maintains a tighter control of gene expression during early infection than during late infection in as much as early genes are highly expressed and late genes show low or no expression; during late infection the total mRNA levels increase, which results in a greater change of absolute late mRNA levels but lower relative levels of late mRNAs.

In order to stringently analyze differential expression in ASFV, we identified the genes which showed the same patterns of differential expression according to separate DESeq2 analyses of the CAGE-seq and RNA-seq data sets. This minimizes any potential biases from each of these complementing techniques. A total of 101 genes showed significant differential expression according to both independent techniques, and the changes in expression levels were significantly correlated between these genes (Spearman’s rank correlation coefficient, ρ = 0.73) (Fig. 6d). Only a small number of genes, 10 out of 101, showed a discrepancy between the two methods (DP63R, I329L, NP419L, B66L, A224L, E248R, O174L, D345L, C315R, and NP1450L), leaving 91 genes confidently classified as early (36) and late (55) genes. Table S6 provides details of these 91 genes and their functions and indicates whether they were previously detected in viral particles (20). The 91 genes with correlated differential expression levels were assigned functional categories based on their annotations in the VOCS database (38) complemented by those of ASFVdb (39) (Fig. 6e). Around one-fifth of early and late genes were classified as uncharacterized, without any functional predictions. The transition between 5 h and 16 h postinfection is characterized by a significant upregulation of genes important for viral morphology and structure, but also the overall diversity of differentially expressed genes changed. A significant difference was seen in the multigene family members; they constitute nearly half of the early genes, but only one (MGF 505-2R) is found among late genes. ORFs annotated as having a transmembrane region (TR) or a putative signal peptide (PSP) were also overrepresented in late infection (Fisher’s test, *P < *0.05). They remain poorly characterized beyond a domain prediction, and 9 proteins (out of 12) of these ORFs could be detected in BA71V virions by mass spectrometry (20).

### Architecture of ASFV gene promoters and consensus elements.

**FIG 7:**
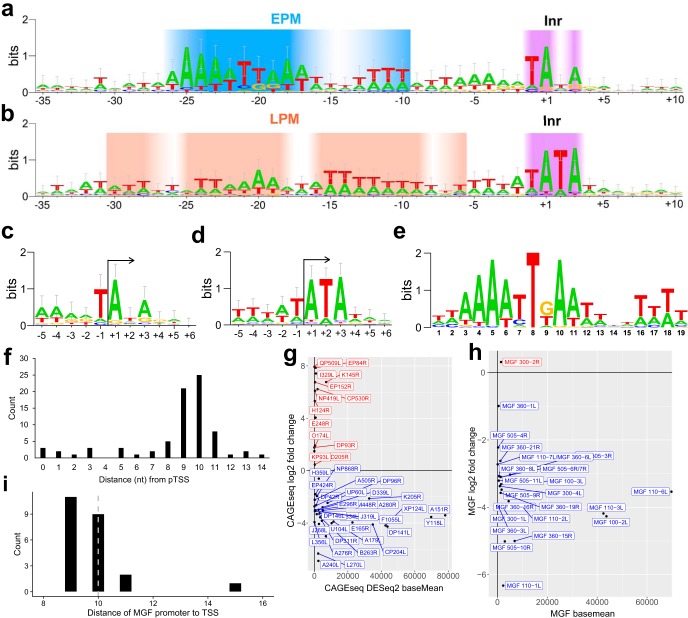
Initiator and promoter sequence signatures of ASFV genes. (a and b) WebLogo 3 (86, 87) of aligned early and late sequences, respectively, surrounding the Inr (+1) from −35 to +10, with gradients representing the base pair conservation of the EPM (blue-white), Inr (purple-white), and LPM (peach-white). (c and d) WebLogo 3 consensus motif with error bars of the 36 early and 55 late gene sequences, respectively, surrounding their respective pTSSs (5 nt up- and downstream), i.e., initiator (Inr) motif. (e) EPM located upstream of all 36 of our classified early genes according to MEME motif search (E value, 8.2e−021); FIMO with a threshold *P* value of <1.0e−4 then identified at least one iteration of this motif upstream of 81 ASFV genes. (f) Distances of the EPM motif 3′ end (nt 19) relative to those of the 78 pTSSs (alternative pTSSs excluded) (4). (g) Expression profiles from DESeq2 analysis (log_2_ fold change versus DESeq2 basemean expression) of genes with only an EPM from the FIMO search of 60 bp upstream of pTSSs. Genes for which FIMO detected both EPM and LPM upstream of pTSSs were excluded. Genes shown in blue demonstrated a negative log_2_ fold change (early genes), and those shown in red demonstrated a positive log_2_ fold change (regardless of significance). (h) Expression profiles as described for panel g for the 26 MGFs where an EPM was detected upstream. (i) Distances of the EPM motif 3′ end (nt 19) relative to those of the MGF pTSSs.

The genome-wide TSS map combined with information about the differential temporal utilization of TSSs allowed us to analyze the sequence context of TSSs and thereby characterize the consensus motifs and promoter architecture of our clearly defined 36 early and 55 late genes. Eukaryotic RNA Pol II core promoters are characterized by a plethora of motifs, including TATA boxes and B recognition elements (BREs) and the initiator (Inr). The first two interact with initiation factors TBP and TFIIB, while the last interacts with RNA Pol II (40). Alignment of regions immediately surrounding pTSSs in the BA71V genome revealed several interesting ASFV promoter signatures; the Inr element overlapping the TSS is a feature that distinguishes between early and late gene promoters (Fig. 7a and b, respectively). The early gene Inr is a TA(+1)NA tetranucleotide motif (where N has no nucleotide preference +1) (Fig. 7c), while the late gene Inr shows a strong preference for the sequence TA(+1)TA (Fig. 7d) that is not to be confused with the TBP-binding TATA box. Our late Inr consensus motif is in good agreement with motifs of 20 previously characterized late gene TSSs (10, 25). To search for additional promoter elements that likely interact with transcription initiation factors, we extended our search to include sequences up to 40 bp upstream of the TSS. Analysis with MEME and FIMO software (41, 42) identified and located a significant 19-nt motif (Fig. 7e) located ∼10 bp upstream of pTSSs for 36 (out of 36) early gene promoter sequences (Fig. 7f), which we have called the early promoter motif (EPM). Our EPM is related to the VACV early gene promoter motif (upstream control element, or UCE) (43, 44) as well as the Kluyveromyces lactis virus-like element (VLE) promoters (45). However, the EPM is not limited to the 36 early genes since a FIMO software (42) motif search identified the EPM within 60 bp upstream of a much larger subset of 81 TSS/TUs, including pNGs and alternative pTSSs, four of which were the early alternative pTSS for I243L, B169L, J154L, and CP80R. Importantly, the limited distance distribution between the EPM and TSS is indicative of constraints defined by distinct protein-DNA interactions, e.g., by transcription initiation factors binding upstream of the TSS and ASFV-RNAP engaging with promoter DNA and TSS (Fig. 7f). Figure 7g illustrates expression profiles of all genes with an EPM upstream according to FIMO, with the majority showing a negative log_2_ fold change between 5 h and 16 h. Since MGF members were overrepresented as early genes (Fig. 6e), we searched directly for the EPM among the FIMO hits. A total of 23 of the 29 MGF members with mapped pTSSs were associated with the EPM element, including consistent early expression and spacing relative to their TSSs (Fig. 7h and i), which suggests that MGF genes are under the control of their own promoters.

**FIG 8:**
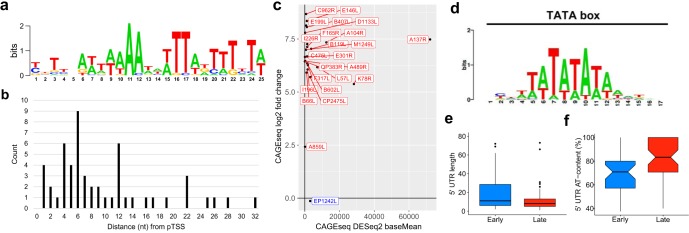
Promoter motif upstream of ASFV late genes. (a) The LPM detected upstream of 17 of our classified late genes from a MEME motif search (E value, 1.6e−003). (b) Distances from a FIMO search (threshold *P* value of <1.0e−4) identified the LPM upstream of 53 ASFV genes (excluding those with alternative pTSSs). Motif distances from pTSSs are represented. (c) Expression profiles as in Fig. 7g and h of genes with only an LPM from the FIMO search of 60 bp upstream of pTSSs. (d) The eukaryotic TATA box motif which was one of 28 hits in a TomTom search of the LPM. (e) 5′ UTR lengths in nucleotides of the 91 early (mean, 39; median, 14) or late (mean, 25; median, 9) classified ASFV genes, starting from the most upstream pTSS (in the case of alternating pTSSs) until the first ATG start codon nucleotide, represented. Nine genes with 5′ UTRs above 80 nt were excluded from the box plot: QP509L (92 nt long), pNG2 (105 nt), I267L (110 nt), B318L (118 nt), C44L (131 nt), DP141L (165 nt), pNG1 (223 nt), EP402R (242 nt), and A118R (332 nt). (f) Percentage AT content of early (mean, 69.0 %; median, 70.9%) and late (mean, 81.7%; median, 83.3%) 5′ UTRs, omitting those of 0 length.

Using the same approach, we searched for promoter sequence motifs associated with late genes. MEME identified a conserved motif upstream of only 17 (out 55) late genes, which we called the late promoter motif (LPM) (Fig. 8a). The spacing (4 to 12 bp) between the LPM and TSS shows a much greater diversity than that of the EPM (Fig. 8b) though genes with the LPM were consistently upregulated (Fig. 8c). A TomTom (46) search identified the LPM motif as a match for 28 distinct motifs, including the canonical TATA box (*P* value of 2.85e−03; E value of 5.16e+00) (Fig. 8d). However, this was not a strong hit, and these motifs bear only a limited resemblance to each other except for their AT-rich biases.

### ASFV mRNAs have 5′ leader regions.

**FIG 9:**
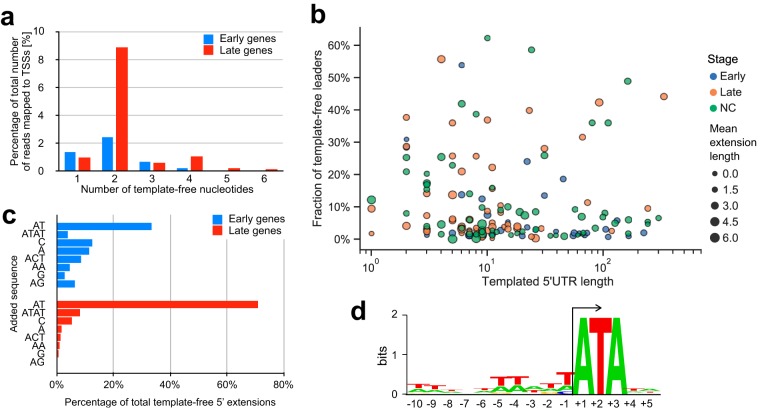
Investigating ASFV-RNAP slippage. (a) Frequency of different lengths of template-free extensions in early- and late-stage samples. (b) Relationship between the length of templated 5′ UTRs and fraction of template-free extensions. Gene 5′ UTRs were split into 36 early (blue), 55 late (orange), and not classified (NC, green) groups. (c) Frequency of most common template-free extensions in the early- and late-stage samples. (d) Sequence logo of region surrounding TSSs of AU- and AUAU-extended transcripts.

Early and late genes in ASFV vary with regard to the length of 5′ untranslated regions (UTRs), i.e., the distance between the 5′ mRNA end and the translation start codon. The 5′ UTRs of late genes are significantly shorter and have a higher AT content than early genes (*P* value of <0.05) (Fig. 8e and f). Surprisingly, a subset of late gene CAGE-seq reads extended upstream of the assigned TSSs and were not complementary to the DNA template strand sequence. In order to rule out any mapping artifacts, we trimmed the CAGE-seq reads by removing the upstream 25 nt and aligned them to the genome at the 5′ boundary of the reads. This did not significantly impair the mapping statistics but highlighted that nearly half of the annotated TSSs (74/158) among both early and late genes are associated with mRNAs that have short 5′ extensions (or leaders), including seven genes with multiple TSSs (Table S7). Most 5′ leaders consist of two or four nucleotides (Fig. 9a), and the presence of the 5′ leaders was not correlated with early or late expression (Fig. 9b). The most common sequence motifs in sequencing reads are AT (33% and 71% of early and late genes, respectively) and ATAT (7% in late genes) (Fig. 9c). In order to investigate any potential sequence dependency of the mRNAs associated with AU-5′ and AUAU-5′ leaders, we scrutinized the template DNA sequence downstream of the TSS and found that all TUs contained the motif ATA at positions +1 to +3 (Fig. 9d). This suggests that the formation of AU leaders is generated by RNA polymerase slippage on the first two nucleotides of the initial A(+1)TANNN template sequence, generating AUA(+1)UANNN or AUAUA(+1)UANNN mRNAs. A different but related slippage has been observed in the VACV transcription system where all postreplicative mRNAs contain short poly(A) leaders which are associated with a consensus Inr TAAAT motif (28).

### Transcription termination of ASFV-RNAP.

**FIG 10:**
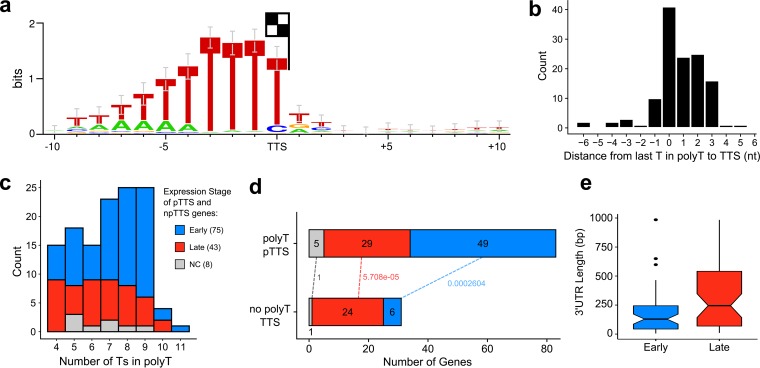
ASFV transcription termination. (a) WebLogo 3 motif of 10 nt upstream and 10 downstream of all pTTS and npTTSs with a poly(T) upstream with ≥4 consecutive Ts, based on 126 TTSs. (b) Distance from 3′ terminal T in poly(T) motif to the TTS (median). (c) The distribution of poly(T) lengths among 126 poly(T) TTSs (median, 7), split into expression stages according to CAGE-seq differential expression analysis (NC, not classified), showing that late gene poly(T)s are shorter (Wilcoxon rank sum test, *P* value of 0.0216). (d) Distribution of gene expression types among the 83 poly(T) pTTSs and 31 non-poly(T) pTTSs. Labels on dotted lines indicate Fisher’s test *P* values of gene types between the two pTTS types, classified from CAGE-seq data. (e) Lengths of 55 early and 53 late gene 3′ UTRs from the stop codon to pTTS (Wilcoxon rank sum test, *P* value of 0.003).

Previous mapping of mRNA 3′ ends has revealed a conserved sequence motif consisting of ≥7 thymidylate residues in the template, which is consistent with 3′ end formation via transcription termination, similar to that of the RNA polymerase III paradigm (16, 47). To investigate the genome-wide sequence context of ASFV transcription termination, we used 3′ RNA-seq sequencing to obtain the sequences immediately preceding ASFV mRNA poly(A) tails, generating a complete map of mRNA 3′ end peaks (Fig. 2a). Using an approach similar to pTSS mapping, CAGEfightR detected a total of 657 termination site clusters and 212 TTSs within 1,000 bp downstream of 1 to 3 ORFs. Because multiple ORFs had more than one cluster within that region (Table S8), we defined 114 primary TTSs (pTTS) as the TTSs with the highest CAGEfightR score in closest proximity to a stop codon; we classified the 98 remaining peaks as nonprimary TTSs (npTTS). We identified a highly conserved poly(T) signal within 10 bp upstream of 126 TTSs (83 pTTSs and 43 npTTSs) that was characterized by ≥4 consecutive T residues (Fig. 10a), with the ultimate residue located on, or 2 bp upstream of, the ultimate T residue in the motif (Fig. 10b). The remaining 86 TTSs were not associated with any recognizable sequence motif besides a single T residue 1 bp upstream of the TTS. Our results are in good agreement with a previous S1 nuclease mapping of 6 coding mRNAs but less so with 17 proposed TTSs which were predicted based on transcript length estimates relative to upstream transcription start sites (Table S2). This may be because only ≥7 consecutive Ts in the template were included to serve as terminators. Our results demonstrate that the total number of consecutive Ts of the poly(T) motif can vary, with poly(T) tracts of CAGE-early genes being longer than those of late genes (Fig. 10c). Finally, we observed differences between CAGE-early and CAGE-late gene termination in as much as poly(T) terminators were overrepresented in CAGE-early and underrepresented in CAGE-late genes (Fig. 10d). The 3′ UTRs (i.e., nucleotide length from translation stop codon to pTTS) of CAGE-late genes were significantly longer than those of CAGE-early genes (Fig. 10e), in good agreement with previous studies on a small number of mRNAs which showed that ASFV transcripts tended to be longer and more variable in length during late infection (Table S2). ORFs are spaced closely in the ASFV genome, and scrutiny of RNA-seq reads revealed a limited extent of transcription readthrough from upstream ORFs into downstream ORFs, likely due to leaky termination (G. Cackett and F. Werner, unpublished observations). However, any additional downstream ORFs generated aberrantly by transcription readthrough would not be able to be translated since there is no evidence of ASFV utilizing internal ribosome entry sites (IRES) that would be required to enable cap-independent translation (7).

## DISCUSSION

Here, we report the first comprehensive ASFV transcriptome study at single-nucleotide resolution. The mapping of 158 TSS and 114 TTS for 159 ASFV genes allowed us to reannotate the BA71V genome. Our results provide detailed information about differential gene expression during early and late infection, the sequence motifs for early and late gene promoters (EPM and LPM and Inr elements) and terminators [poly(T) motif], and evidence of quasitemplated AU RNA-5′ tailing by the ASFV-RNAP.

We have discovered seven novel putative genes, some of which are highly conserved with the aggressively virulent strains (Georgia 2007/1 and Belgium 2018/1) that have caused the current outbreak in Europe (Table 3). This suggests that BA71V has more genes in common with its virulent cousins than initially thought.

Our results demonstrate that the majority of ASFV genes show some degree of differential expression from early to late infection (Fig. 1). Interestingly, our CAGE-seq results demonstrate that early genes are expressed at higher levels during early infection than late genes during late infection (Fig. 6a to c). Future experiments including spike-in controls are needed to confidently quantify the absolute mRNA levels during early and late infection (48). The RNA sequencing methods used here quantify the steady-state RNA levels and not RNA synthesis rates, and without information about ASFV mRNA stability, it is not possible to distinguish between early mRNAs retained until late infection and early genes being transcribed at later stages. Nascent ASFV mRNA synthesis rates and half-lives could be determined using techniques including transient transcriptome sequencing (TT-seq) (49) or by using transcription inhibitors including actinomycin D (50). Frustratingly, many of the highly expressed genes are uncharacterized (Fig. 5a). These gene products are important candidates for further functional characterization and may emerge as promising targets for vaccine development.

We have shown that MGFs show distinct downregulation from early to late infection, while genes annotated as transmembrane region or putative signal peptide genes (though poorly characterized beyond this), along with structural or viral morphology genes, are overrepresented in late infection (Fig. 6e). Our CAGE analysis also identified TSS signals unlikely to serve as primary TSSs for annotated genes (Fig. 3a; see also Table S9 in the supplemental material); these could provide a rich hunting ground for small noncoding RNAs (sncRNAs). One TSS cluster associated with an sncRNA gene (at position 71302 on the BA71V genome) was previously reported by Dunn et al. (51) as ASFVsRNA2, that is encoded in the antisense orientation relative to that of the ASFV RNA polymerase subunit RPB6-encoding gene. Further investigation of antisense sncRNAs in the BA71V transcriptome may uncover further examples of riboregulation, i.e., a more complex method of modulating its own or host gene expression beyond the protein level.

While eukaryotic Pol II and archaeal RNAP critically rely on initiation factors TBP and TFIIB for transcription initiation on all mRNA genes, bacterial RNAP obtains specificity for subsets of gene promoters by associating with distinct sigma factors (52). ASFV-RNAP is related to archaeal and eukaryotic RNA polymerases; detailed phylogenetic analyses reveal that the RPB1 subunit is most closely related to the RNA polymerase I homologue (3, 45, 53). However, transcription initiation of early and late genes appears to be directed by two distinct sets of general initiation factors and their cognate DNA recognition motifs, as our TSS mapping demonstrates. The first feature of all ASFV promoters is the Inr element, a tetranucleotide motif overlapping the TSS with an A residue serving as the initiating nucleotide, similar to most RNAP systems. The similarity of early and late gene Inr sequences is likely because the Inr makes sequence-specific contacts with amino acid side chains of the two largest RNAP subunits (RPB1 and RPB2). The EPM and LPM are located upstream of the TSS; both are AT rich though distinct in sequence (Fig. 7e and 8a). The distance distribution of EPM is narrow (located 9 to 10 bp upstream of the TSS) while the distance between the LPM and TSS shows greater variation and is located closer (4 to 6 bp) to the TSS. The high sequence and distance conservation of the EPM, especially exemplified for early expressed MGFs (Fig. 7h and i), emphasizes the EPM’s role in tight control of transcription during early infection. Considering the close relationship between ASFV and VACV, we posit that the EPM is recognized by a heterodimeric ASFV-BA71V D1133L/G1340L initiation factor (VACV D6/A7) (11), consistent with the late expression of these genes (Fig. 6d) (54). Presence of D1133L/G1340L gene products along with RNAP in viral particles (20) provides a system that is primed to initiate ASFV transcription of early genes.

ASFV-TBP (B263R) is an early gene, and ASFV-TFIIB (C315R) is expressed throughout infection. We propose that the LPM is utilized by ASFV-TBP and -TFIIB homologues, neither of which was detected in virions (20). A functional comparison of the LPM to the classical Pol II core promoter elements, BRE/TATA box, is compelling. However, the tight spacing between the LPM and TSS is incompatible with the overall topology of a classical eukaryotic and archaeal TATA-TBP-TFIIB-RNA Pol II preinitiation complex (PIC), where the BRE/TATA promoter elements are located ∼24 bp upstream of the TSS (55). Considering low sequence conservation between cellular and ASFV TBP (8) and unusual spacing of the LPM and Inr, the structure of ASFV LPM-TBP-TFIIB-RNAP PIC is likely very different from canonical RNA Pol II PICs. Additionally, factors including ASFV B175L and B385R may contribute to the PIC, as was proposed for VACV A1 and A2 (56, 57). At this stage, we cannot rule out a limited overlap between early and late genes without additional information, including insights into pre- and postreplicative gene expression patterns, mRNA stability of early and late genes, and knowledge about all regulatory factors that enable the temporal regulation of ASFV transcription. To unequivocally attribute factors to their cognate binding motifs genome-wide, a chromatin immunoprecipitation (ChIP) approach is required; the results may be full of surprises and have the potential to shed light on multistage gene expression patterns, including the possibility of a more complex promoter architecture where some genes are under the control of more than one promoter.

An in-depth characterization of the global gene regulation in ASFV with a higher temporal resolution is essential to assess how closely ASFV follows the cascade-like patterns of VACV (11). While two genes have been proposed to be intermediate genes in ASFV, both of them are also expressed during intermediate and late stages (I226R) and during early, intermediate, and late stages (I243L). Thus, there is no hard evidence of genes that are specifically expressed during the intermediate stage (26). A combination of a reversible replication inhibitor and a conditionally regulated late transcription factor has been successfully used to characterize intermediate gene expression in VACV (58). Such an approach might also be useful to identify intermediate ASFV genes and would help us refine the LPM that in our current analysis could reflect a combination of late and intermediate gene promoters.

We found several examples of alternative, gene-internal, TSS utilization with the potential to increase the complexity of the viral proteome; protein variants may provide the means to generate distinct functionalities, which has also been described in VACV by Yang et al. (28). Our TSS mapping uncovered a form of transcript slippage by the ASFV-RNAP occurring on promoters that start with an A(+1)TA motif, where mRNAs are extended by one or two copies of the dinucleotide AU. This is reminiscent of VACV, where late gene transcripts containing a poly(A) 5′ UTR (28) are associated with improved translation efficiency and reduced reliance on cap-dependent translation initiation (59, 60); similarly, distinct functional attributes of poly(A) leaders in translation have been documented in eukaryotes (61). Whether the 5′ AU- and AUAU-tailing is a peculiarity of the ASFV-RNAP initiation or whether these mRNA 5′ leaders have any functional implications remains to be investigated. The structural determinants underlying RNAP slippage are interactions between the template DNA sequence and the RNAP and/or transcription initiation factors; the differential use of distinct initiation factors for the transcription of early and late ASFV genes may account for differences in leader sequences.

The mechanisms underlying transcription termination of multisubunit RNAP are diverse (62, 63). Our analyses of genome-wide ASFV RNA-3′ ends allowed the mapping of the ASFV terminome. Over half of mRNA 3′ ends are characterized by a stretch of seven U residues, with the TTS mostly coinciding with the last T residue in the template DNA motif, which is in good agreement with ASFV terminators that have been individually mapped (15, 16). In contrast, VACV appears to utilize a motif ∼40 nt upstream of the mRNA 3′ ends (64, 65). In essence, the ASFV-RNAP is akin to archaeal RNAPs and RNA Pol III, where a poly(U) stretch is the sole *cis*-acting motif without any RNA secondary structures characteristic of bacterial intrinsic terminators (63). The pTTSs without any association with poly(U) motifs are still likely to represent bona fide termination sites since RNA-seq reads were decreasing toward these termination sites, despite no clear conserved sequence motif. However, ASFV does encode several (VACV-related) RNA helicases that have been speculated to facilitate transcription termination and/or mRNA release (10, 66). Future functional studies will address the molecular mechanisms of termination, including the role of putative termination factors.

Understanding the molecular mechanisms of the ASFV transcription system is not only of academic interest. Unless effective vaccines in conjunction with antiviral treatments against ASFV are developed, a large proportion of the global pig population is projected to die in the context of this terrible disease (World Organisation for Animal Health/OIE [https://www.oie.int]). The rational design of drugs that target the gene expression machinery is crucially reliant on our knowledge about the ASFV-RNAP, the basal factors that govern its function, and the DNA sequences they interact with, while vaccine development benefits from the intricate knowledge about gene expression patterns. Our results directly contribute to these burning issues for animal husbandry.

## MATERIALS AND METHODS

### RNA sample extraction from Vero cells infected with BA71V.

Vero cells (catalog no. 84113001; Sigma-Aldrich) were grown in six-well plates, infected in two replicate wells for 5 h or 16 h at a multiplicity of infection of 5 with the ASFV BA71V strain, and collected in TRIzol lysis reagent (Thermo Fisher Scientific) separately after growth medium was removed. Infected cells were collected at 5 h postinfection (samples for RNA-seq: S3-5h and S4-5h; CAGE-seq, S1-5h and S2-5h; 3′ RNA-seq, E-5h_1 and E-5h_1) and at 16 h postinfection (RNA-seq, S5-16h and S6-16h; CAGE-seq, S3-16h and S4-16h; and 3′ RNA-seq, L-16h_1 and L-16h_1). RNA was extracted according to the manufacturer’s instructions for TRIzol extraction, and the subsequent RNA-pellets were resuspended in 50 μl of RNase-free water and DNase treated (Turbo DNAfree kit; Invitrogen). RNA quality was assessed via a Bioanalyzer (Agilent 2100) before ethanol precipitation. For CAGE-seq and 3′ RNA-seq, samples were sent to CAGE-seq (Kabushiki Kaisha DNAFORM, Japan) and Cambridge Genomic Services (Department of Pathology, University of Cambridge, Cambridge, UK), respectively.

### RNA-seq, CAGE-seq, and 3′ RNA-seq library preparations and sequencing.

For RNA-seq, samples were resuspended in 100 μl of RNase-free water and poly(A) enriched using a NEXTflex poly(A) beads kit (Bioo Scientific) according to the manufacturer’s instructions, and quality was assessed via a Bioanalyzer. A NEXTflex Rapid Directional qRNA-Seq kit was utilized to produce paired-end indexed cDNA libraries from the poly(A)-enriched RNA samples, according to the manufacturer’s instructions. Per-sample cDNA library concentrations were calculated via a Bioanalyzer and Qubit fluorometric quantitation (Thermo Fisher Scientific). Sample S3-5h, S4-5h, S5-16h, and S6-16h cDNA libraries were twice separately sequenced on an Illumina MiSeq instrument, generating 75-bp reads (see Table S1 in the supplemental material) and 12 FASTQ files.

Library preparation and CAGE-seq of RNA samples S1-5h, S2-5h, S3-16h, and S4-16h was carried out by CAGE-seq (Kabushiki Kaisha DNAFORM, Japan). Library preparation produced single-end indexed cDNA libraries for sequencing; in brief, this included reverse transcription with random primers and oxidation and biotinylation of 5′ mRNA cap, followed by RNase One treatment removing RNA not protected in a cDNA-RNA hybrid. Two rounds of cap-trapping were performed using streptavidin beads, washing away uncapped RNA-cDNA hybrids. Next, RNase One and RNase H treatment degraded any remaining RNA, and cDNA strands were subsequently released from the streptavidin beads and quality assessed via Bioanalyzer. Single-strand index linker and 3′ linker were ligated to released cDNA strands, and primer containing an Illumina sequencer priming site was used for second-strand synthesis. Samples were sequenced using an Illumina NextSeq 500 platform producing 76-bp reads (Table S1).

3′ RNA-seq was carried out with samples E-5h_1, E-5h_2, L-16h_1, and L-16h_2 using a Lexogen QuantSeq 3′ mRNA-Seq Library Prep kit FWD for Illumina according to the manufacturer’s instructions. Library preparation and sequencing were carried out at Cambridge Genomic Services (Department of Pathology, University of Cambridge, Cambridge, UK) on a single NextSeq flow cell producing 150 bp reads (Table S1).

### Sequencing quality checks and mapping to ASFV and Vero genomes.

FastQC (67) analysis was carried out on all FASTQ files; for RNA-seq, FASTQ files were uploaded to the web platform Galaxy (https://usegalaxy.org) (68, 69), and all reads were trimmed by the first 10 and last 1 nt using FASTQ Trimmer (70). After read trimming, FASTQ files originating from the same RNA samples were concatenated. RNA-seq reads were mapped to the ASFV-BA71V (GenBank accession no. NC_001659.2) and Vero cell (from African green monkey; NCBI RefSeq no. GCF_000409795.2) genomes using Bowtie 2 directly after trimming (27), with alignments output in SAM file format. FASTQ-analyzed CAGE-seq reads showed consistent read quality across the 76-bp reads, except for nucleotide 1. This was an indicator of the 5′ mRNA methylguanosine due to the reverse transcriptase used in library preparation (71); therefore, the reads were mapped in their entirety to the ASFV-BA71V (GenBank accession no. U18466.2) and Vero cell (NCBI RefSeq GCF_000409795.2) genomes.

FASTQC-analyzed 3′ RNA-seq reads showed relatively varying and poorer quality after nucleotide 65. Cutadapt (72) was utilized to extract only fastq reads with 18 consecutive As at the 3′ end followed by the sample i7 Illumina adapter, selecting only for reads containing the 3′ mRNA end and the poly(A) tail. The 18 A-adapter sequences were then trimmed, and FASTQC-analyzed reads were mapped via Bowtie 2 to ASFV-BA71V (GenBank accession no. U18466.2) and Vero cell (NCBI RefSeq GCF_000409795.2) genomes.

### CAGE analysis and TSS mapping.

CAGE-seq-mapped sample BAM files were converted to BigWig (BW) format with BEDtools genomecov (73) to produce per-strand BW files of 5′ read ends. Stranded BW files were input for TSS prediction in RStudio (74) with the Bioconductor (75) package CAGEfightR (76). Genomic feature locations were imported as a TxDb object from the GenBank U18466.2 genome gene feature file (GFF3), modified to include C44L (12). CAGEfightR was used to quantify the CAGE tag transcripts mapping at base pair resolution to the ASFV-BA71V genome at CAGE TSSs (CTSSs). CTSS values were normalized by tags per million for each sample and pooled, and only CTSSs supported by presence in ≥2 samples were kept. CTSSs were assigned to clusters, with merging of CTSSs within 50 bp of one another, filtering out pooled TPM-normalized CTSS counts below 25, and assigned a “thick” value as the highest CTSS peak within that cluster. CTSS clusters were assigned to annotated GenBank U18466.2 ORFs (if clusters were between 300 bp upstream and 200 bp downstream of an ORF). Clusters were classified tssUpstream if located within 300 bp upstream of an ORF, proximal if located within 500 bp of an ORF, coding DNA sequence (CDS) if within the ORF, not available (NA) if no annotated ORF was within these regions (excepting pNG), and antisense if within these regions but antisense relative to the ORF.

Cluster classification was not successful in all cases; therefore, manual adjustment was necessary. Integrative Genomics Viewer (IGV) (77) was used to visualize BW files relative to the BA71V ORFs, and incorrectly classified clusters were corrected. Clusters with the tssUpstream classification were split into subsets for each ORF. The primary cluster subset contained either the highest scoring CAGEfightR cluster or the highest scoring manually annotated peak, and the highest peak coordinate was defined as the primary TSS (pTSS) for an ORF. Further clusters associated with these ORFs were classified as nonprimary, and the highest peak was classified as a nonprimary TSS (npTSS).

If the strongest CTSS location was intra-ORF and corroborated with RNA-seq coverage, then the ORF was redefined as starting from the next ATG downstream. For the 28 intergenic CTSSs, IGV was used to visualize if CAGE BW peaks were followed by RNA-seq coverage downstream and whether the transcribed region encoded a putative ORF by using NCBI Open Reading Frame Finder (78).

### TTS mapping.

TTSs were mapped in a similar manner to TSSs, and CAGEfightR was utilized as described above to locate clusters of 3′ RNA-seq peaks though the method differed in some respects: input BigWig files contained the 3′ read-end coverage extracted from BAM files using BEDtools genomecov. Clusters were detected for the 3′ RNA-seq peaks in the same manner as before, except that clusters < 25 nt apart were merged, giving a total of detected 567 clusters. BEDtools was used to check whether the highest point of each cluster (TTS) was within 500 bp or 1,000 bp downstream of annotated ORFs and pNGs. TTSs were then filtered out if 10 nt downstream of the 3′ end had ≥ 50% As to exclude clusters potentially originated from miss-priming. TTS clusters for pNG3 and pNG4 were initially filtered out but included in the final 212 TTSs due to their strong RNA-seq agreement. In cases of multiple TTS clusters per gene, we defined the highest CAGEfightR-scored one within 1,000 bp downstream of ORFs as the primary (pTTS) unless no clear RNA-seq coverage was shown or manually annotated from the literature for O61R (15).

### DESeq2 differential expression analysis of ASFV genes.

A new GFF was produced for investigating differential expression of ASFV genes across the genome with changes from the original GFF from GenBank accession number U18466.2. For all 151 ASFV ORFs which had identified pTSSs, we defined their transcription units as beginning from the pTSS coordinate to the ORF end. Since no pTSS was identified for ORFs E66L and C62L, these entries were left as ORFs within the GFF, while the seven putative pNGs were defined as their pTSSs down to the genome coordinate at which the RNA-seq coverage ends. In eight cases in which genes had alternative pTSSs for the different time points, the TUs were defined as the most upstream pTSS down to the ORF end. For analyzing differential expression with the CAGE-seq data set, a GFF was created with BEDtools extending from the pTSS coordinate 25 bp upstream and 75 bp downstream; however, in cases of alternating pTSSs, this TU was defined as 25 bp upstream of the most upstream pTSS and 75 bp downstream of the most downstream pTSS. HTSeq-count (79) was used to count reads mapping to genomic regions described above for both the RNA- and CAGE-seq sample data sets. The raw read counts were then used to analyze differential expression across these regions between the time points using DESeq2 (default normalization described by Love et al. [80]), and the regions showing changes with an adjusted *P* value (padj) of <0.05 were considered significant. Further analysis of ASFV genes used their characterized or predicted functions as found in the VOCS tool database (https://4virology.net/) (38, 81) or ASFVdb (39) entries for the ASFV-BA71V genome.

### Early and late promoter analysis.

DESeq2 results were used to categorize ASFV genes into two simple subclasses: early, i.e., genes downregulated from early to late infection, and late, i.e., those genes upregulated from early to late infection. For the genes with newly annotated pTSSs (151 including 7 pNGs but excluding 15 alternative pTSSs), sequences 30 bp upstream and 5 bp downstream were extracted from the ASFV-BA71V genome in FASTA format using BEDtools. The 36 early and 55 late genes and all 166 pTSSs (including alternative ones) at once were analyzed using MEME software (http://meme-suite.org) (82), searching for 5 motifs with a width of 10 to 25 nt (other settings at default). Significant motifs (E value of <0.05) detected via MEME were submitted to a following FIMO (42) search (*P* value cutoff of < 0.0001) of 60 nt upstream of the total 166 pTSS sequences (including pNGs and alternative pTSSs), and TomTom software (46) search (name, UP00029_1; database, uniprobe_mouse) to find similar known motifs.

### Data availability.

Sequencing data from RNA-seq, CAGE-seq, and 3′ RNA-seq are available in the NCBI Sequence Read Archive (SRA) under BioProject accession number PRJNA590857. The processed data for two replicates are visualized in an UCSC Genome Browser (83) and can be accessed at https://bit.ly/2TazQxK. The tracks include corrected gene annotations (primary TSSs, primary TTSs, and ORF coordinates), raw coverage of 5′ ends (CAGE-seq) and 3′ ends (3′-RNA-seq), and reads per kilobase per million (RPKM) values for the RNA-seq data. Coverage for the forward and reverse strands are shown in blue and red, respectively.

Results from differential gene expression analysis with DESeq2 of CAGE-seq and RNA-seq are found in Tables S4 and S5, respectively. The 91 genes showing the same patterns of differential expression according to both of these NGS techniques are found in Table S6. Details of nontemplated extensions detected from CAGE-seq are in Table S7. CAGEfightR-detected cluster peaks from 3′ RNA-seq after removal of those arriving from poly(A) miss-priming are described in Table S8. All 779 CAGEfightR-detected cluster peaks from CAGE-seq are listed in Table S9.

## Supplementary Material

Supplemental file 1

Supplemental file 2

Supplemental file 3

Supplemental file 4

Supplemental file 5

Supplemental file 6

Supplemental file 7

Supplemental file 8

Supplemental file 9

Supplemental file 10
